# Euphorbiasteroid Abrogates EGFR and Wnt/β-Catenin Signaling in Non-Small-Cell Lung Cancer Cells to Impart Anticancer Activity

**DOI:** 10.3390/molecules27123824

**Published:** 2022-06-14

**Authors:** Na Young Kim, Chakrabhavi Dhananjaya Mohan, Arunachalam Chinnathambi, Sulaiman Ali Alharbi, Gautam Sethi, Kanchugarakoppal S. Rangappa, Kwang Seok Ahn

**Affiliations:** 1 Department of Science in Korean Medicine, Kyung Hee University, 24 Kyungheedae-ro, Dongdaemun-gu, Seoul 02447, Korea; nay0kim@naver.com; 2 Department of Studies in Molecular Biology, University of Mysore, Manasagangotri, Mysore 570006, India; cd.mohan@yahoo.com; 3 Department of Botany and Microbiology, College of Science, King Saud University, Riyadh 11451, Saudi Arabia; carunachalam@ksu.edu.sa (A.C.); sharbi@ksu.edu.sa (S.A.A.); 4 Department of Pharmacology, Yong Loo Lin School of Medicine, National University of Singapore, Singapore 117600, Singapore; 5 Institution of Excellence, Vijnana Bhavan, University of Mysore, Manasagangotri, Mysore 570006, India

**Keywords:** NSCLC, euphorbiasteroid, GSK-3β, EGFR, Wnt/β-catenin

## Abstract

EGFR and Wnt/β-catenin signaling pathways play a prominent role in tumor progression in various human cancers including non-small-cell lung carcinoma (NSCLC). Transactivation and crosstalk between the EGFR and Wnt/β-catenin pathways may contribute to the aggressiveness of cancers. Targeting these oncogenic pathways with small molecules is an attractive approach to counteract various types of cancers. In this study, we demonstrate the effect of euphorbiasteroid (EPBS) on the EGFR and Wnt/β-catenin pathways in NSCLC cells. EPBS induced preferential cytotoxicity toward A549 (wildtype EGFR-expressing) cells over PC-9 (mutant EGFR-expressing) cells. EPBS suppressed the expression of EGFR, Wnt3a, β-catenin, and FZD-1, and the reduction in β-catenin levels was found to be mediated through the activation of GSK-3β. EPBS reduced the phosphorylation of GSK-3β^S9^ with a parallel increase in β-TrCP and phosphorylation of GSK-3β^Y216^. Lithium chloride treatment increased the phosphorylation of GSK-3β^S9^ and nuclear localization of β-catenin, whereas EPBS reverted these effects. Forced expression or depletion of EGFR in NSCLC cells increased or decreased the levels of Wnt3a, β-catenin, and FZD-1, respectively. Overall, EPBS abrogates EGFR and Wnt/β-catenin pathways to impart its anticancer activity in NSCLC cells.

## 1. Introduction

Lung cancer stands first in terms of global cancer-related deaths with a mortality rate of 1.8 million deaths in 2020 as per the statistics of the World Health Organization [1]. Non-small-cell lung carcinoma (NSCLC) and small-cell lung carcinoma (SCLC) are the two major types of lung cancer [2,3]. NSCLC contributes approximately 80–85% of total lung cancer cases, while 10–15% is contributed by SCLC [4,5]. Smoking, exposure to carcinogens (radon and asbestos), occupational hazards, and family history are the major risk factors associated with the development and progression of NSCLC [6,7,8,9,10,11]. Current treatment strategies involve surgical therapies including sleeve resection, lobectomy, and pneumonectomy [12,13], as well as nonsurgical therapies such as radiofrequency ablation, radiotherapy, chemotherapy, and immunotherapy [14,15,16,17]. Delayed diagnosis often results in poor clinical outcomes. Despite the continuous efforts and advancements in the development of therapeutic agents against NSCLC, current chemotherapeutic treatment strategies are marginally effective in the prognosis and curing of NSCLC. Erlotinib, afatinib, and gefitinib are quinazolamine-based small-molecule inhibitors of EGFR, which have been approved for the treatment of metastatic NSCLC [18,19,20]. In addition, panitumumab, cetuximab, brigatinib, and icotinib are other approved drugs targeting EGFR for the treatment of various types of cancers [21]. However, studies have shown that most patients develop resistance to erlotinib, gefitinib, and other first-generation drugs [22]. Therefore, it is the need of the hour to develop new therapeutic agents against oncogenic signaling pathways that are activated in NSCLC cells.

The Wnt signaling pathway plays a central role in the development of cancers and is a critical regulator of cell-cell interactions, cell fate, and migration [23]. The absence of Wnt ligand on the frizzled (FZD) receptor, results in the formation of a complex comprising Disheveled, Axin, APC, GSK-3β, and β-catenin, in which GSK-3β phosphorylates and β-TrCP (an E3 ubiquitin ligase) ubiquitylates β-catenin to promote its proteasome-mediated degradation [24,25]. As a result, the nuclear localization of β-catenin is inhibited, which leads to the suppression of target gene expression. The presence of Wnt ligand with FZD receptor and its coreceptor (LRP: low-density lipoprotein receptor-related protein) complex results in the sequestration of GSK-3β and blockage of phosphorylation of β-catenin. Stabilized β-catenin translocates into the nucleus and interacts with other transcription factors to induce the expression of genes associated with cell proliferation, migration, and other functions. Additionally, EGFR signaling independently regulates cell growth, migration, and epithelial-mesenchymal transition. Many studies have demonstrated the crosstalk between EGFR and Wnt signaling pathways [26,27,28]. The binding of Wnt ligands to the FZD receptor has been demonstrated to transactivate via matrix metalloproteinase-mediated release of soluble EGFR ligands, such as transforming growth factor α [29]. Notably, there is a positive correlation between EGFR activation due to EGFR mutation and the increased nuclear translocation ability of β-catenin in NSCLC [30]. Therefore, targeting the Wnt signaling pathway could be an ideal strategy to block the proliferation of cancer cells.

Natural compounds obtained from various sources such as plants, bacteria, and fungi are serving as an inexhaustible source of anticancer compounds [31,32,33,34]. Many of the secondary metabolites derived from mother nature have been used clinically for the treatment of various human diseases [35,36,37,38]. Among the natural compounds, triterpenoids are known to have good anticancer activity [39]. Recently, Zhu and colleagues investigated the anticancer activity of tricyclic diterpenoids isolated from *Azadirachta indica*, and most of the investigated tricyclic diterpenoids displayed good cytotoxicity toward lung cancer cells [40]. Similarly, multiple reports also suggest that tricyclic diterpenoids are endowed with good anticancer activity. Therefore, we selected euphorbiasteroid (EPBS), a tricyclic terpenoid found in *Euphorbia lathyris* L., whose cytotoxic activity and mechanism of action are not precisely elucidated in lung cancer cells. During the literature survey, we used the keyword “euphorbiasteroid” and searched in Pubmed and found only two results (last accessed on 7 June 2022) indicating that researchers have not extensively explored the anticancer activity of EPBS. Choi and colleagues reported that EPBS has the potential to reverse P-glycoprotein-mediated multidrug resistance in human sarcoma cells [41]. Park and colleagues demonstrated that EPBS suppresses adipogenesis of 3T3-L1 cells by activating the AMPK pathway [42]. It has also been shown to elevate the Fas/FasL pathway to induce apoptosis in leukemia cells [43]. Despite being discovered long ago, there are no concrete reports to demonstrate the mode of action of EPBS in inducing cytotoxicity in cancer cells. It is important to note that A549 cells have been extensively used as an appropriate cell type to study the effect of small molecules on EGFR-dependent signaling pathways. Our initial studies using EPBS on NSCLC cells displayed some degree of cytotoxicity; therefore, we further investigated the effect of EPBS on Wnt/β-catenin and EGFR signaling pathways (which are constitutively active) in NSCLC cells.

## 2. Results

### 2.1. EPBS Has Preferential Cytotoxicity toward A549 Cells over PC-9 Cells

**Figure 1:**
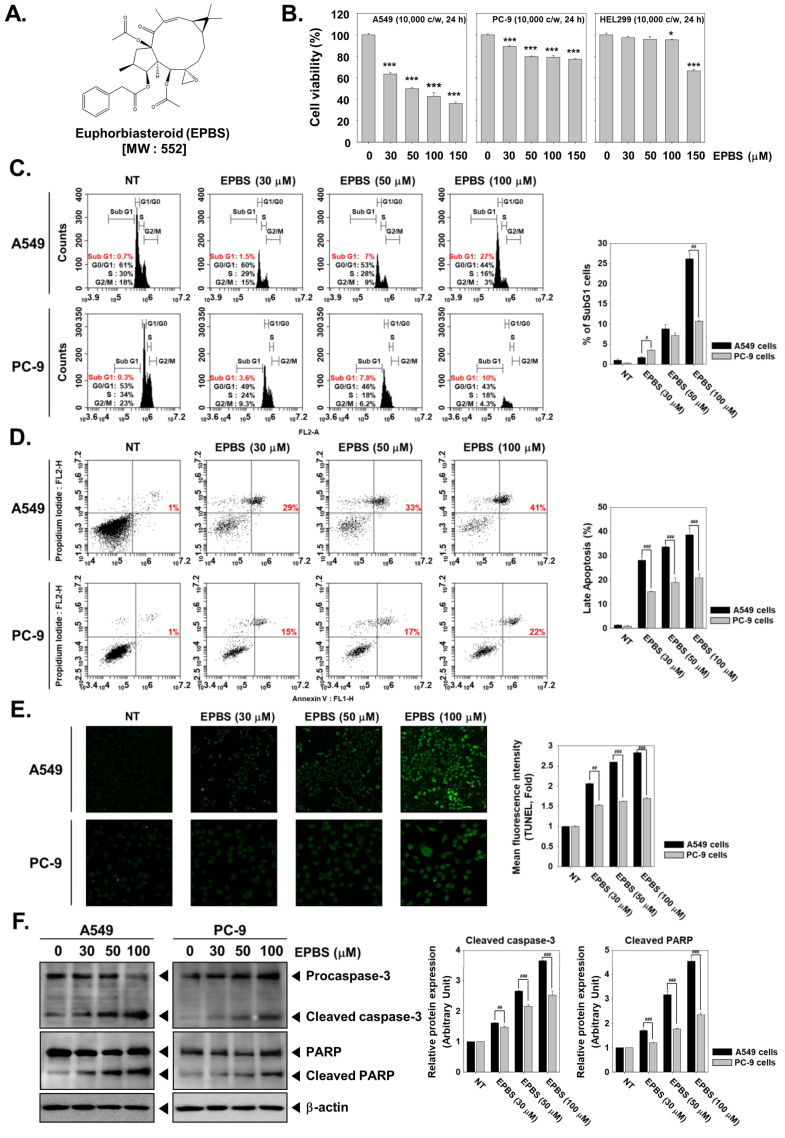
EPBS induces apoptosis and increases the subG1 cell population in NSCLC cells. (**A**) The chemical structure of EPBS. (**B**) Human NSCLC A549 cells, PC-9 cells, and human lung normal HEL299 cells (1 × 10^4^ cells/well) were treated with EPBS (0, 30, 50, 100, and 150 µM) for 24 h. For examining the cell viability, an MTT assay was performed. *** *p* < 0.001 and * *p* < 0.05 vs. nontreated (NT) cells. (**C**) A549 cells and PC-9 cells (5 × 10^5^ cells/well) were treated with EPBS (0, 30, 50, and 100 µM for 24 h) and collected and then digested with RNase A for 1 h. Both cells were stained with propidium iodide, and cell-cycle arrest was analyzed with flow cytometry. (**D**) EPBS (0, 30, 50, and 100 µM for 24 h)-treated A549 and PC-9 cells were collected and stained with FITC/PI for 15 min at RT. The stained cells were analyzed with flow cytometry. (**E**) A total of 2 × 10^4^ cells/well of both cells were seeded on eight-well chamber plates. The indicated concentration of EPBS was treated for 24 h and then fixed with 4% PFA. After 20 min, the fixed cells were permeable with 0.2% Triton X-100 for 10 min. The cells were stained with a TUNEL staining reagent. Results were analyzed by a confocal microscope. (**F**) The cells were treated with EPBS for 24 h, and then Western blot analysis was performed. ^###^ *p* < 0.001, ^##^ *p* < 0.01, and ^#^ *p* < 0.05 for A549 cells vs. PC-9 cells. All experiments were performed at least three times, and representative data are shown.

The chemical structure of EPBS is provided in Figure 1A. We performed an MTT assay to evaluate whether different concentrations (0, 30, 50, 100, and 150 µM) of EPBS can have a cytotoxic effect on NSCLC (A549 and PC-9) cell lines. We found a dose-dependent decrease in the viability of A549 cells of 100%, 63%, 49%, 42%, and 36% upon treatment with EPBS (0, 30, 50, 100, and 150 µM), respectively. On the other hand, the PC-9 cells showed cell viability of about 100%, 89%, 80%, 79%, and 77% at the indicated concentrations, respectively (Figure 1B). EPBS did not present a substantial cytotoxic effect on non-diseased lung fibroblast HEL299 cells.

### 2.2. EPBS Increases the SubG1 Cell Population in NSCLC Cells

During apoptosis, caspase-3 gets activated, which results in the activation of caspase-dependent DNases, which in turn fragment the genomic DNA into oligonucleotides [44,45]. The cells with decreased DNA are termed hypodiploid cells which are detected as SubG1 cells in flow cytometric analysis. We performed flow cytometric analysis to examine the effect of EPBS on the cell-cycle distribution of A549 and PC-9 cells. There was an increase in the subG1 population (0.7%, 1.5%, 7%, and 27%) of A549 cells at different concentrations of EPBS (0, 30, 50, and 100 µM), respectively, indicating that cancer cells are driven to death after treatment with EPBS (Figure 1C). However, PC-9 cells responded feebly to EPBS treatment.

### 2.3. EPBS Induces Caspase-Mediated Apoptosis in NSCLC Cells

We next performed annexin V/propidium iodide staining to investigate whether the cell death observed upon EPBS treatment in flow cytometric analysis is due to apoptosis. We noted a substantial increase in the number of late apoptotic cells (1%, 29%, 33%, and 41%) at different concentrations of EPBS treatment (0, 30, 50, and 100 µM) in A549 cells respectively, suggesting that EPBS induces apoptosis-mediated cell death (Figure 1D). We next performed a TUNEL assay to ensure that cells were undergoing apoptosis upon EPBS treatment, and these results were also in agreement with the observations of the annexin V/propidium iodide assay (Figure 1E). The responsiveness of PC-9 cells to EPBS was low in the annexin V/propidium iodide staining and TUNEL assay. Next, we analyzed the levels of procaspase-3, full-length PARP, and their cleaved fragments. We observed an evident decrease in procaspase-3 and full-length PARP with a parallel corresponding increase in their cleaved fragments (Figure 1F). The results of annexin V/propidium iodide staining, TUNEL assay, and Western blotting of caspase-3 and PARP concluded that EPBS induces apoptosis in NSCLC cells.

### 2.4. EPBS Downregulates the Expression of EGFR and Wnt/β-Catenin Pathway Proteins in NSCLC Cells

**Figure 2:**
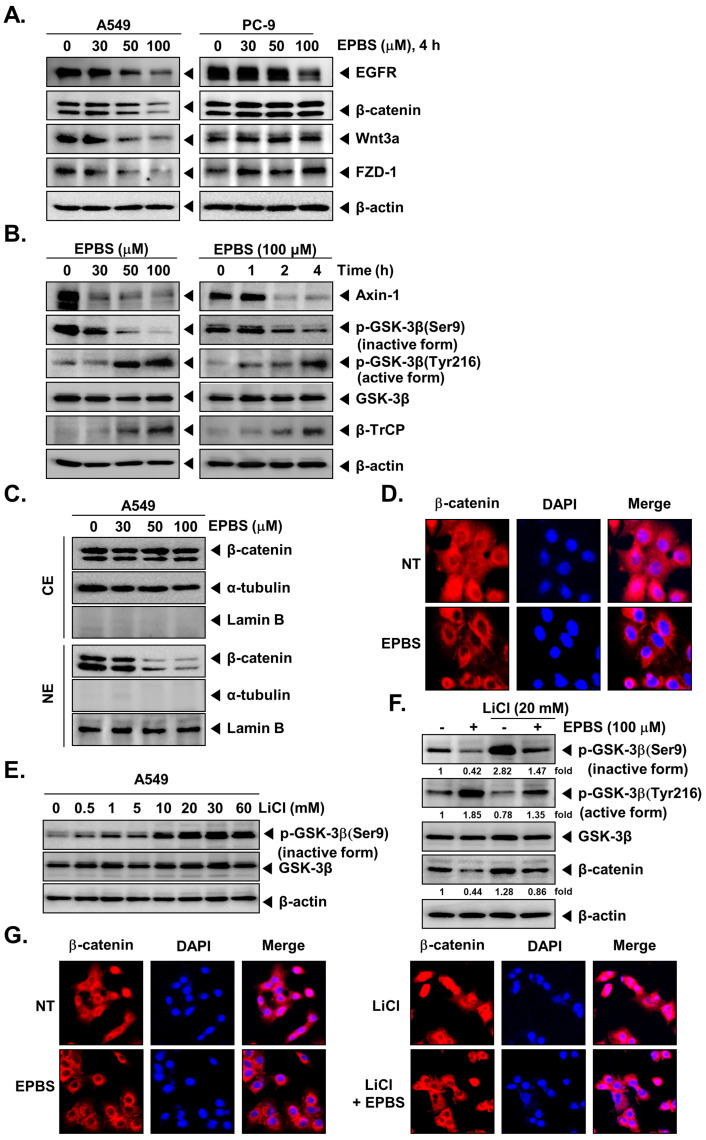
EPBS downregulates EGFR and the Wnt/β-catenin pathway and decreases the nuclear pool of β-catenin. (**A**) A549 cells and PC-9 cells (5 × 10^5^ cells/well) were treated with EPBS (0, 30, 50, and 100 µM) for 4 h. Western blot analysis was performed. For confirming equal protein loading, the same blot membrane was stripped and re-probed with a β-actin antibody. (**B**) A549 cells (5 × 10^5^ cells/well) were treated with the indicated concentration of EPBS for 4 h or with 100 µM of EPBS for indicated time intervals. EPBS-treated cells were collected and lysed, and then an equal amount of lysates were detected by Western blot analysis. (**C**) EPBS (0, 30, 50, and 100 µM for 4 h)-treated A549 cells (5 × 10^5^ cells/well) were collected, and then cytoplasmic extracts (CE) and nuclear extracts (NE) were prepared. As loading control of cytoplasmic extracts or nuclear extracts, α-tubulin or lamin B antibodies were used. (**D**) A549 cells were treated with EPBS (100 µM) for 4 h, and β-catenin translocation was analyzed by immunocytochemistry. (**E**) A549 cells were treated with the indicated concentration of LiCl (inhibitor of GSK-3β) for 4 h, and then Western blotting was carried out. (**F**,**G**) A549 cells were treated with 20 mM of LiCl and 100 µM of EPBS for 4 h. Then, Western blot analysis and immunocytochemistry were performed. The fold values at the bottom of the blots indicate the fold activation for the untreated control group. Representative data of at least three independent experiments are shown.

Next, we investigated the effect of EPBS on the expression of EGFR and proteins of the Wnt/β-catenin signaling pathway in A549 and PC-9 cells. We observed a concentration-dependent significant decrease in the expression of EGFR, β-catenin, Wnt3a, and FZD-1 in A549 cells, and the change in the expression of these proteins was not evident in PC-9 cells (Figure 2A). In PC-9 cells, only EGFR expression was downmodulated upon treatment with EPBS, and proteins of the Wnt/β-catenin pathway remained unaltered. Since PC-9 cells were low responders to EPBS treatment, we considered A549 cells for further experiments.

### 2.5. EPBS Modulates the Activity of GSK-3β in NSCLC Cells

We next examined the effect of EPBS on the expression of Axin-1 and β-transducin repeat-containing protein (β-TrCP), and phosphorylation of GSK-3β^S9 and Y216^. The phosphorylation of GSK-3β at S9 and Y216 is an indicator of the inactive and active forms of GSK-3β, respectively. We found that EPBS treatment decreased the expression of Axin-1 and increased that of β-TrCP in A549 cells (Figure 2B). Furthermore, EPBS decreased the phosphorylation of GSK-3β^S9^ with a parallel increase in the phosphorylation of GSK-3β^Y216^ in a time- and dosage-dependent fashion (Figure 2B).

### 2.6. EPBS Decreases the Nuclear Pool of β-Catenin in NSCLC Cells

We further inquired whether EPBS has any effect on the nuclear localization of β-catenin by Western blotting analysis in A549 cells. A drastic dose-dependent reduction was found in the nuclear levels of β-catenin in EPBS-treated cells (Figure 2C). α-Tubulin and lamin B were used as internal controls of cytoplasmic and nuclear extracts. The distribution of β-catenin in cytoplasm and nucleus upon EPBS treatment was further verified using immunofluorescence analysis. EPBS substantially downregulated the nuclear pool of β-catenin (Figure 2D), and these observations were on par with the results of the Western blotting analysis.

### 2.7. EPBS Reverses the Lithium Chloride (LiCl)-Induced Inhibition of GSK-3β in NSCLC Cells

Increased phosphorylation of GSK-3β^S9^ implies the inhibition of GSK-3β. LiCl is an inducer of GSK-3β^S9^ phosphorylation, thereby serving as an inhibitor of GSK-3β activity. We treated A549 cells with different concentrations of LiCl and analyzed phosphorylation of GSK-3β^S9^ using Western blotting analysis. Upon LiCl treatment, the phosphorylation intensity of GSK-3β^S9^ was maximum at 20 mM (Figure 2E); therefore, the same dose of LiCl was used to analyze the effect of EPBS on LiCl-induced GSK-3β^S9^ phosphorylation. LiCl treatment significantly increased the GSK-3β^S9^ phosphorylation compared to the basal level, whereas cotreatment of LiCl and EPBS counteracted the LiCl-induced GSK-3β^S9^ phosphorylation (Figure 2F). Increased phosphorylation of GSK-3β^Y216^ implies the activation of GSK-3β. LiCl treatment decreased the phosphorylation of GSK-3β^Y216^, and cotreatment with EPBS reversed this effect (Figure 2F).

### 2.8. EPBS Decreases the LiCl Induced Nuclear Localization of β-Catenin in NSCLC Cells

The results of Western blotting were supported by the results of immunofluorescence studies where LiCl treatment increased the nuclear localization of β-catenin over basal levels, whereas the cotreatment with EPBS substantially declined this effect (Figure 2G). DAPI was used to stain the nucleus to produce overlay images.

### 2.9. EPBS Mitigates Wnt/β-Catenin Signaling Cascade in EGFR-Overexpressing NSCLC Cells

**Figure 3:**
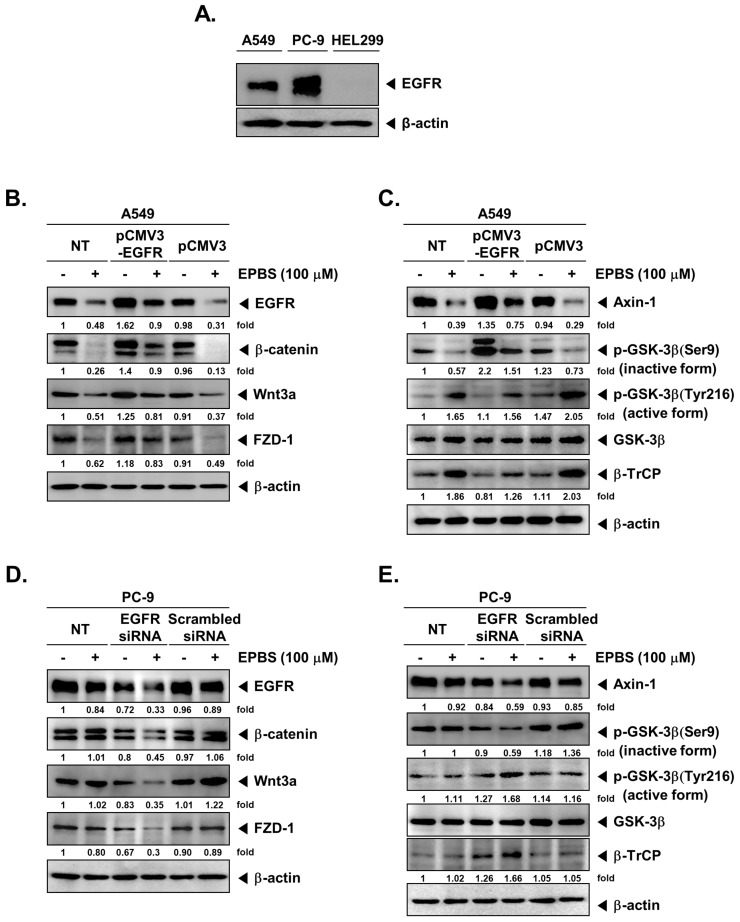
EPBS mitigates the Wnt/β-catenin signaling pathway in EGFR−overexpressing or EGFR−knockdown NSCLC cells. (**A**) Human NSCLC A549 and PC-9 cells and human normal lung HEL299 cells were seeded on six-well plates (5 × 10^5^ cells/well), and then cells were harvested and lysed. Western blot analysis was carried out. (**B**,**C**) A549 cells were seeded on 24-well plates (8 × 10^4^ cells/well). A549 cells were transfected with 300 ng of human EGFR/HER1 gene ORF cDNA clone expression plasmid (pCMV3-EGFR) or pCMV3-untagged negative control vector (pCMV3) with transfection reagent in serum-free medium (SFM) for 24 h. After 24 h, the transfected A549 cells were changed with a complete medium and then treated with 100 μM of EPBS for 4 h. Western Blot analysis was performed. (**D**,**E**) PC-9 cells were seeded on 24-well plates (8 × 10^4^ cells/well) overnight and were transfected with 100 nM of EGFR siRNA or scrambled siRNA transfection reagent in a serum-free medium for 36 h. The transfected PC-9 cells were treated with 100 µM of EPBS in a completed medium. After 4 h, the cells were harvested and lysed, and then Western Blot analysis was performed. The fold numbers at the bottom of the blots indicate fold activation for the nontreated control group. All experiments were performed at least three times, and representative data are shown.

We analyzed the expression of EGFR in A549, PC-9, and HEL299 cells using Western blotting analysis and found that PC-9 cells had a higher amount of EGFR compared to A549 cells (Figure 3A). We next overexpressed EGFR in A549 cells by transfecting them with pCMV3-EGFR and examined the effect of EPBS on the expression of EGFR, β-catenin, Wnt3a, and FZD-1. Induced expression of EGFR enhanced the levels of β-catenin, Wnt3a, and FZD-1, whereas EPBS treatment mitigated these effects, indicating that EPBS abrogates constitutive activation of the Wnt/β-catenin signaling cascade in A549 cells (Figure 3B).

### 2.10. EPBS Modulates the Activity of GSK-3β in EGFR-Overexpressing NSCLC Cells

We next examined the effect of EPBS on the expression of Axin-1 and β-TrCP, as well as on the phosphorylation of GSK-3β^S9 and Y216^ in A549 cells-transfected with pCMV3-EGFR. The transfection of A549 cells with pCMV3-EGFR elevated the expression of Axin-1 and phosphorylation of GSK-3β^S9^ with a parallel decrease in β-TrCP (Figure 3C). EPBS treatment reversed the expression and phosphorylation of proteins that were altered upon transfection, indicating that EPBS can also interfere with the forced expression/activation of Wnt/β-catenin pathway proteins (Figure 3C).

### 2.11. EPBS Modulates the Expression and Activity of Wnt/β-Catenin Pathway Proteins in EGFR-Knockdown NSCLC Cells

We next depleted the EGFR using EGFR-siRNA in PC-9 (EGFR-overexpressing) cells and tested the effect of EPBS on the expression of the Wnt/β-catenin pathway proteins. When EGFR siRNA (100 nM) was transfected into the PC-9 cells (36 h), the expression of EGFR was reduced (Figure 3D). There was no significant change in the levels of EGFR, Wnt/β-catenin pathway proteins upon EPBS exposure in non-transfected or scrambled siRNA transfected PC-9 cells (Figure 3D). In contrast, PC-9 cells transfected with EGFR-siRNA upon EPBS treatment downregulated the expression of Wnt/β-catenin proteins such as β-catenin, Wnt3a, FZD-1, Axin-1, and inactive form of GSK-3β, with a parallel increase in the expression of the active form of GSK-3β and β-TrCP (Figure 3E).

### 2.12. Overexpression and Knockdown of EGFR Modulates Apoptosis in NSCLC Cells

**Figure 4:**
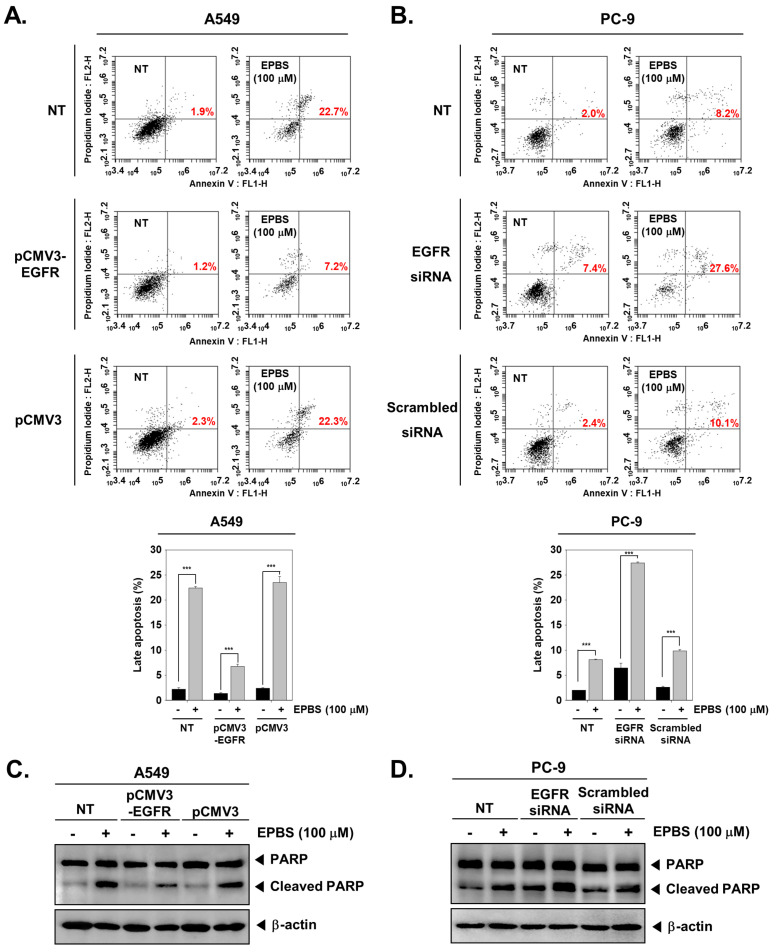
EPBS induces apoptosis in overexpression and knockdown of EGFR in NSCLC cells. (**A**) A total of 8 × 10^4^ cells/well of A549 cells were seeded on 24-well plates overnight and then transfected with pCMV3−EGFR or pCMV3 plasmid (300 ng) in a serum-free medium for 24 h. Then, 100 µM of EPBS was applied for 24 h, and cells were collected. (**B**) PC-9 cells (8 × 10^4^ cells/well) were transfected with EGFR siRNA or scrambled siRNA (100 nM) for 36 h. The transfected cells were treated with 100 µM EPBS for 24 h, and then the cells were collected. Both collected cells were reacted with FITC-tagged annexin V antibodies and PI for 15 min at RT. The stained cells were analyzed by a flow cytometer. *** *p* < 0.001 vs. EPBS-nontreated cells. (**C**) A549 cells were transfected with pCMV3−EGFR or pCMV3 plasmid for 24 h and then treated with 100 µM of EPBS for 24 h. Western blot analysis was carried out. (**D**) PC-9 cells were transfected with EGFR siRNA or scrambled siRNA for 36 h. The transfected PC-9 cells were treated with 100 µM EPBS for 24 h, and then Western blot analysis was performed for analyzing the expression of PARP protein. After at least three experiments were performed, the representative data were selected and shown.

Furthermore, we analyzed whether EPBS can induce apoptosis in EGFR-overexpressing and EGFR-depleted NSCLC cells using the annexin V/propidium iodide assay. For this, A549 cells were transfected with pCMV3-EGFR to cause overexpression of EGFR, which was followed by EPBS treatment and measurement of apoptosis. Non-transfected cells treated with EPBS served as the control. The percentage of apoptotic cells was 22.7% in EPBS-treated non-transfected cells, whereas it decreased to 7.2% in EPBS-treated plasmid-transfected cells (Figure 4A). Similarly, we depleted EGFR in PC-9 cells by transfecting with EGFR-siRNA followed by EPBS treatment and measurement of apoptosis. Non-transfected cells treated with EPBS served as the control. The percentage of apoptotic cells was 7.4% in EGFR-knockdown PC-9 cells which was increased to 27.6% in EPBS-treated EGFR-siRNA transfected cells, whereas it was decreased to 10.1% in EPBS-treated non-transfected cells (Figure 4B). A similar trend in the expression of full-length PARP and its cleaved fragment was observed upon overexpression and knockdown of EGFR in A549 and PC-9 cells, respectively (Figure 4C,D). These results indicate the critical involvement of EGFR in the apoptosis-inducing effect of EPBS in NSCLC cells.

## 3. Discussion

The EGFR pathway is one of the prominent signaling cascades studied in relevance to the progression of human cancers, and it has been reported to be elevated in many types of cancers including NSCLC [46,47,48]. Targeting EGFR has been clinically successful and largely implemented in the treatment of lung cancer. EGFR is an attractive therapeutic target in the treatment of NSCLC, as the overexpression of EGFR is observed in up to 80% of NSCLC patients [49,50]. Although studies have reported the correlation between overexpression of EGFR and dismal prognosis, the definitive role of EGFR in the prognosis of NSCLC remains unclear [51]. Several small-molecule EGFR tyrosine kinase inhibitors have been approved for the treatment of NSCLC patients. The first-, second-, and third-generation tyrosine kinase inhibitors such as erlotinib, afatinib, and osimertinib, respectively, have been approved for treating NSCLC patients harboring EGFR-activating mutations [52]. However, most patients gradually develop resistance to the first-generation drugs [22]. Therefore, it is essential to discover new small molecules that either has a direct inhibitory effect on EGFR or block other oncogenic signaling cascades that crosstalk with the EGFR pathway. Few reports have suggested the crosstalk between EGFR and Wnt/β-catenin pathways [26,27,28]. The interaction of Wnt with the FZD receptor has been shown to release transforming growth factor α, which is a known ligand of EGFR activation [29]. Lee and colleagues demonstrated that EGFR modulates the localization, stability, and transcriptional activity of β-catenin in oral cancers [53]. They proposed that EGFR triggers the phosphorylation of GSK-3β^S9^, which leads to the inhibition of its kinase activity and the suppression of phosphorylation-dependent proteasome-mediated degradation of β-catenin [53]. Additionally, the phosphorylation of β-catenin induced by EGFR is believed to dissociate β-catenin from the membrane and promote its translocation into the nucleus [53]. It has also been noted that there is a positive correlation between EGFR activation and increased nuclear translocation of β-catenin in NSCLC [30]. The convergence between Wnt/β-catenin and EGFR signaling in cancers has previously been thoroughly reviewed [54,55]. Importantly, activation of the Wnt/β-catenin pathway has been noted in various types of human cancers. In the present report, we investigated the effect of EPBS on the expression and activation state of EGFR and Wnt/β-catenin pathway proteins.

We initially investigated the cytotoxic potential of EPBS against wildtype A549 cells and EGFR-mutant (amplification) PC-9 cells. Interestingly, the results of the cytotoxicity assay presented that the A549 cells were high responders and PC-3 cells were low responders to EPBS treatment. The reason for the low responsiveness of PC-9 cells could be due to the presence of EGFR-activating mutation (deletion of exon 19 in EGFR) [56,57,58]. Further analysis revealed that EPBS markedly suppressed the levels of EGFR, β-catenin, Wnt3a, and FZD-1 in A549 cells, whereas the effect of EPBS on the expression of these proteins was feeble in PC-9 cells. On the other hand, phosphorylation of GSK-3β^S9^ was reduced with a parallel increase in phosphorylation of GSK-3β^Y216^ upon EPBS treatment. Hwang and colleagues also reported the decrease in expression of Wnt3a and β-catenin, and the increase in expression of phosphorylated GSK-3β^Y216^ in MDA-MB-231 cells when treated with moracin D [59]. They concluded that blockade of the Wnt3a/FOXM1/β-catenin axis and activation of GSK-3β is essential for inducing apoptosis in breast cancer cells [59]. The phosphorylation of GSK-3β^S9^ inactivates it under the control of upstream kinases such as protein kinase A, Akt, protein kinase C, p90 ribosomal S6 kinase/MAPK-activating protein, and p70 ribosomal S6 kinase. The phosphorylation of GSK-3β^Y216^ is under the control of Src-family tyrosine kinases (Fyn and MEK1) [60]. Nakata and colleagues proposed that targeting the Akt/β-catenin pathway can enhance the therapeutic efficacy of EGFR tyrosine kinase inhibitors in drug-resistant cancers, suggesting the crucial role of β-catenin in cancer progression [61]. Our results demonstrated that nuclear translocation of β-catenin is significantly reduced upon EPBS treatment, suggesting that it may be implemented alongside EGFR tyrosine kinase inhibitors in drug-resistant cancers.

LiCl is an inhibitor of GSK-3β and has been reported to serve as an enhancer of activation of Wnt signaling [62]. LiCl induced the phosphorylation of GSK-3β^S9^ and EPBS treatment reversed this effect, indicating that EPBS promotes the activation of GSK-3β with a parallel decline in the levels of β-catenin. These results indicate that EPBS triggers the activation of GSK-3β, which in turn induce the degradation of β-catenin. To establish the link between EGFR and the Wnt/β-catenin pathway, we overexpressed and depleted EGFR in A549 and PC-9 cells using pCMV3-EGFR and EGFR-siRNA, respectively. Overexpression of EGFR led to the increased expression of Wnt3a, FZD-1, and β-catenin, and depletion of EGFR led to the decreased expression of Wnt3a, FZD-1, and β-catenin. In another set of experiments, overexpression of EGFR led to the activation of GSK-3β^S9^ (an inactive form of GSK-3β) and decreased expression of β-TrCP and GSK-3β ^Y216^ (an active form of GSK-3β), while depletion of EGFR resulted in activation of GSK-3β activity and β-TrCP. In total, EPBS treatment reversed the changes that took place upon overexpression of EGFR, and EPBS treatment triggered more effects of the changes that took place upon depletion of EGFR. These results suggest that EPBS can modulate the activity of Wnt/β-catenin pathway proteins in both EGFR-overexpressing and EGFR-depleted cancer cells. Overall, we present EPBS as a novel inhibitor of the EGFR and Wnt/β-catenin signaling pathways in NSCLC cells.

## 4. Materials and Methods

### 4.1. Reagents

Euphorbiasteroid (Catalog No. CFN90641) was purchased from ChemFaces (Wuhan, Hubei, PRC). Fetal bovine serum (FBS), DMEM, and RPMI-1640 medium were procured from Thermo Scientific HyClone (Waltham, MA, USA). Alexa Fluor^TM^ 594 donkey anti-rabbit IgG (H + L) antibody was procured from Invitrogen (Eugene, OR, USA). Antibodies against EGFR, β-catenin, Axin-1, GSK-3β (Ser9), GSK-3β (Tyr216), GSK-3β, cleaved caspase-3, and PARP were purchased from Cell Signaling Technology (Boston, MA, USA). Antibodies against Wnt3a, FZD-1, β-actin, β-TrCP, α-tubulin, lamin B, and caspase-3 were procured from Santa Cruz Biotechnology (Dallas, TX, USA). Human EGFR/HER1 gene ORF cDNA clone expression plasmid (Cat. No: HG10001-UT) and pCMV3-untagged negative control vector (Cat. No: CV011) were obtained from Sino Biological, Inc. (Wayne, PA, USA). EGFR siRNA (Cat. No: sc-29301) was obtained from Santa Cruz Biotechnology (Dallas, TX, USA). The transfection reagent (iNfect^TM^) was purchased from Intron Biotechnology Inc. (Seongnam-si, Gyeonggi-do, Republic of Korea).

### 4.2. Cell Lines and Culture Conditions

Human non-small-cell lung cancer (NSCLC) A549 cells were purchased from American Type Culture Collection (Manassas, VA, USA). Human NSCLC PC-9 cells were procured from Immuno-Biological Laboratories (Gunma, Japan). A549 cells were propagated in low-glucose DMEM, and the PC-9 cells were propagated in the RPMI-1640 medium. Both media contained 1% P/S and 10% inactivated FBS. A549 and PC-9 cells were maintained at 37 °C in 5% CO_2_.

### 4.3. Cell Viability Assay

The MTT assay was performed for analyzing the cytotoxic effect of EPBS, as previously described [35]. A549 and PC-9 cells (1 × 10^4^ cells/well) were seeded on a 96-well plate and incubated with different concentrations of EPBS (0, 30, 50, 100, and 150 µM) for 24 h. Thereafter, cells were treated with MTT solution (2 mg/mL, 30 µL/well) for 2 h followed by the addition of lysis buffer (100 µL/well) overnight. The absorbance of the resultant solution was examined at 570 nm using a VARIOSKAN LUX (Thermo Fisher, Waltham, MA, USA) [63].

### 4.4. Western Blotting

Western blotting was performed to determine the protein expression as described previously [64]. A549 cells and PC-9 cells were seeded on six-well plates and then incubated overnight at 37 °C. The cells were treated with EPBS with given concentrations for indicated time intervals. The EPBS-treated cells were harvested and lysed, equal protein amounts were prepared using the Bradford assay, and Western blotting analysis was carried out as indicated in our previous reports.

### 4.5. Immunocytochemistry for β-Catenin Localization

EPBS-treated A549 cells were fixed with 4% paraformaldehyde for about 20 min at room temperature followed by washing with PBS thrice. Localization of β-catenin was examined as indicated earlier. The cells were treated with 0.2% Triton X-100 (10 min at RT) for permeability and then blocked with 5% BSA for 1 h. The samples were mounted using Fluorescent Mounting Medium (Sigma-Aldrich, St. Louis, MO, USA) followed by visualization using the FluoView FV1000 confocal microscope (Olympus, Tokyo, Japan) [45].

### 4.6. Cell-Cycle Analysis

EPBS (0, 30, 50, and 100 µM)-treated A549 and PC-9 cells were harvested using Trypsin/EDTA and then fixed with cold 70% ethanol overnight at 4 °C, before performing flow cytometric analysis as previously reported [65,66]. Fixed cells were incubated with RNases A (1 mg/mL) for 1 h at 37 °C. After the RNase A reaction, both cells were treated with propidium iodide (PI) and then detected using a BD Accuri^TM^ C6 Plus Flow Cytometer (BD Bioscience, Franklin Lakes, NJ, USA) [67].

### 4.7. Annexin/PI Staining Assay

Both cells were treated with EPBS (0, 30, 50, and 100 µM) for 24 h. The cells were harvested and collected. The collected cells were stained with PI- and FITC-tagged annexin V antibodies for 15 min at RT. The annexin V/PI-stained cells were detected and analyzed using a BD Accuri^TM^ C6 Plus Flow Cytometer (BD Bioscience, Franklin Lakes, NJ, USA).

### 4.8. Terminal Deoxynucleotidyl Transferase-Mediated dUTP Nick End Labeling (TUNEL) Assay

The EPBS-treated A549 and PC-9 cells were fixed with 4% paraformaldehyde for 20 min and then reacted with 0.2% Triton X-100 for 10 min. TUNEL staining was carried out as per the instructions from the manufacturer’s protocol. The samples were mounted with Mounting Medium and then detected using the FluoView FV1000 confocal microscope (Tokyo, Japan).

### 4.9. Transfection of pCMV3-EGFR Vector and pCMV-Untagged Vector in A549 Cells

A549 cells were seeded on 24 well-plate (8 × 10^4^ cells/well). A549 cells were transfected with 300 ng of human EGFR/HER1 gene ORF cDNA clone expression plasmid (pCMV3-EGFR plasmid) or pCMV3-untagged negative control vector (pCMV3 plasmid) for 24 h using iNfect^TM^ in vitro transfection reagent. After transfection, the cells were treated with 100 µM EPBS, and then Western blot analysis and annexin V assay were performed.

### 4.10. Transfection of EGFR siRNA and Scrambled siRNA in PC-9 Cells

PC-9 cells were seeded on a 24-well (8 × 10^4^ cells/well) overnight. PC-9 cells were transfected with 100 nM EGFR siRNA for 36 h. The transfection process was performed by using an iNfect^TM^ in vitro transfection reagent in a serum-free medium. The transfected PC-9 cells were treated with 100 µM EPBS. After transfection, the cells were collected, and then Western blot analysis and annexin V assay were performed as described earlier [68].

### 4.11. Statistical Analysis

All values are presented as the mean ± SD. For determining the statistical significance of the data, Student’s unpaired *t*-test was used. Significance was set at * *p* < 0.05, ** *p* < 0.01, and *** *p* < 0.001 or at ^#^ *p* < 0.05, ^##^ *p* < 0.01, and ^###^ *p* < 0.001.

## Data Availability

Not applicable.

## Sample Availability

Samples of the compounds are not available from the authors.
